# The Influence of Forest Landscape Spaces on Psychological and Visual Attention Responses: An Analysis Based on Different Seasons and Sexes

**DOI:** 10.3390/ijerph23040425

**Published:** 2026-03-29

**Authors:** Soyeon Kim

**Affiliations:** Department of Forest Healing, Catholic Kwandong University, Gangneung 25601, Republic of Korea; neon5947@cku.ac.kr

**Keywords:** eye-tracking, natural landscape, perceived restorativeness scale (PRS), semantic differential (SD) scale, forest healing

## Abstract

**Highlights:**

**Public health relevance—How does this work relate to a public health issue?**

- Rapid urbanization and increased exposure to digital environments have reduced opportunities for contact with natural environments, contributing to public health concerns such as stress, mental fatigue, and reduced psychological well-being. This study examines visual attention patterns toward forest landscapes using eye-tracking technology to better understand how exposure to natural environments supports psychological restoration and emotional stability.
- By applying an area-of-interest (AOI)-based eye-tracking analysis, the study objectively identifies which elements of forest landscapes (e.g., trees, sky, and water) attract visual attention. This approach helps clarify the mechanisms through which natural environments contribute to mental health from a public health perspective.

**Public health significance—Why is this work of significance to public health?**

- This study extends previous findings on the restorative effects of nature by providing quantitative evidence on how specific visual elements of natural environments contribute to stress reduction and psychological recovery.
- The findings offer evidence-based insights for the design of health-promoting environments such as urban parks, forest-healing sites, and therapeutic gardens, thereby supporting public health strategies aimed at improving population mental well-being.

**Public health implications—What are the key implications or messages for practitioners, policy makers and/or researchers in public health?**

- The results suggest that specific landscape components of natural environments can guide visual attention and promote psychological restoration, providing scientific evidence for the development of health-supportive green spaces and forest-based health promotion policies.
- The application of eye-tracking technology introduces an objective method for evaluating nature-based health interventions and may contribute to future public health research on preventive health strategies using natural environments.

**Abstract:**

This study investigated seasonal and sex-based differences in psychological responses and area-of-interest (AOI)-based visual attention, as well as the associations between these variables, using images of the same forest-healing landscape captured in summer and autumn. A total of 40 adults (20 males and 20 females) participated in an eye-tracking experiment combined with psychological assessments, including the Perceived Restorativeness Scale (PRS-11) and semantic differential (SD) evaluations. Mixed-design ANOVA results indicated that perceived restorativeness remained stable across seasons, whereas emotional evaluations were significantly higher in autumn than in summer. Significant interaction effects between season and sex were observed in selected gaze metrics within the sky AOI, while the forest AOI showed a consistent main effect of sex across seasons. Spearman’s correlation analysis revealed a strong positive association between autumn PRS and SD scores, suggesting that aesthetic appreciation contributes to restorative perception. In addition, a significant negative correlation between forest and pond AOIs in autumn indicated a seasonal redistribution of visual attention. These findings highlight the importance of component-level landscape analysis and demonstrate that seasonal variation and user characteristics jointly influence perceptual and attentional responses in forest-healing environments. The results provide empirical implications for evidence-based forest landscape design and seasonal management strategies.

## 1. Introduction

Rapid urbanization and digital-centric lifestyles have led to a decrease in humannature interactions, which can result in an ‘extinction of experience’, subsequently reducing interest in and support for nature [1]. Therefore, the psychological recovery function provided by the natural environment has become an important research topic in terms of emotional and public health outcomes [2].

Previous studies have shown that exposure to greenery and nature is significantly associated with stress relief and promotion of well-being [3,4]. The restorative effects of nature have been explained through the attention restoration theory (ART), which provides a theoretical framework describing how natural environments alleviate cognitive fatigue through restorative properties, such as enchantment and detachment [5,6]. Subsequent studies have reported that visual exposure to natural scenes alone can induce psychological recovery responses and have expanded our understanding of the restorative effects of nature using various methodologies [3,7]. However, many existing studies have focused on average comparisons between natural and urban environments or changes observed before and after visits [2,3]. Therefore, analyses evaluating how specific visual elements within a landscape (sky, forest, pond) influence perceived restorativeness and emotional responses through perceptual processes remain limited.

In the context of forest healing, the landscape is the most rapidly processed stimulus at the beginning of an experience, and visual attention allocation can have a significant impact on emotional evaluation and experience quality. Therefore, it is necessary to quantitatively verify the elements that receive considerable attention by evaluating natural landscapes at the component level [8,9]. Eye-tracking is a method that can overcome these limitations.

Eye-tracking can objectively measure the distribution of visual attention to landscape images using indicators such as gaze and dwell time [9]. Studies have explored the relationship between attention patterns and restorative assessments of natural landscape components through area-of-interest (AOI)-based analysis [10,11,12]. However, natural landscapes can vary according to the season, which can change the prominence of landscape elements and visual attention strategies. Furthermore, because sex differences are potentially present in visual exploration methods and emotional evaluations, individuals of different sexes may respond differently to the same stimulus.

Nevertheless, few studies have integrated and analyzed psychological responses and AOI-based gaze behavior in forest-healing landscapes at the same location, considering both seasonal and sex factors. Therefore, this study comprehensively analyzes the AOI-based gaze behavior (sky, forest, pond), sense of recovery using a Perceived Restorativeness Scale (PRS-11), and semantic differential (SD)-based emotional evaluation among adult males and females using summer and autumn forest-healing landscape images taken at the same location. In addition, this study will investigate the relationship between visual attention allocation and psychological restorative outcomes. Overall, this study aims to provide empirical evidence for the component-level design and evaluation of forest-healing landscapes by identifying seasonal and sex-based differences in psychological responses and visual attention allocation.

Although previous studies have demonstrated the restorative benefits of natural environments, most investigations have relied on global comparisons between natural and urban settings or pre–post exposure designs. Component-level analyses examining how specific landscape elements attract visual attention and contribute to restorative perception remain limited. Furthermore, seasonal variation—an inherent characteristic of forest environments—has rarely been integrated into AOI-based visual attention research. Even fewer studies have simultaneously examined seasonal and sex-based differences in both psychological and gaze responses within the same landscape setting. Furthermore, the distinctive coloration and strong visual contrast typical of autumn deciduous foliage act as key perceptual factors that naturally captivate visual attention and influence positive affective responses. However, when considering the broad applicability of these perceptual dynamics, it is important to note that in many other forest ecosystems, such as evergreen coniferous forests, such seasonal color changes are minimal.

Therefore, this study addresses these research gaps by integrating seasonal variation, sex differences, and AOI-level visual attention analysis within a unified experimental framework.

## 2. Materials and Methods

### 2.1. Participants and Experimental Procedures

#### 2.1.1. Participants

Forty adults (20 males and 20 females) aged 40–70 years participated in this study. Notably, a significant proportion of the participants identified as students. These individuals are mature adult learners enrolled in university programs, which reflects the diverse educational demographic of the 40–70 age group.

All participants were selected based on the absence of visual and/or neurological conditions that could interfere with the eye-tracking experiments, and all had normal or corrected-to-normal vision. Participation was voluntary and all individuals provided written informed consent after receiving a detailed explanation of the research objectives and procedures. This study was conducted in accordance with the ethical standards approved by the Institutional Review Board of Catholic Kwandong University (IRB No. CKU-25-01-0403). To control the order effects related to stimulus presentation, a counterbalanced design was employed, and the sequence of stimulus images was randomized for each participant.

#### 2.1.2. Experimental Stimuli

**Figure 1:**
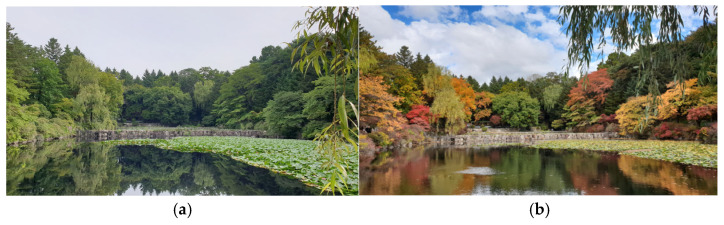
Image taken at the exact location representing summer (**a**) and autumn (**b**) that was selected as the stimulus scene(Photograph taken by the author).

The experiment consisted of summer and autumn natural scenery images taken by a researcher from the same forest landscape location, and a total of two images, one for each season, were used (Figure 1). All images were produced using high-definition digital photographs (Galaxy S4, Samsung, Suwon, Republic of Korea) with a resolution of 1920 × 1080 pixels. These images were selected primarily for scenes where visual differences, such as color, brightness, and texture depending on the season were revealed. Each image was presented to the participants for 30 s. To prevent sequence effects, the two visual stimuli (summer and autumn images) were presented to each participant in a randomized order.

These presentation times and procedures followed experimental designs widely utilized in existing studies to reliably measure gaze behavior and psychological responses to natural landscape images [10,11,13]. The stimuli were selected based on previous research that analyzed visual perception characteristics and emotional responses to natural landscapes [14,15]. In addition, reflecting the seasonal natural landscape preference characteristics and positive effects of waterfront elements reported in previous studies [14,15,16,17], a forest landscape incorporating both forest and water elements was selected as the stimulus image. Furthermore, using static images of a single location was a deliberate methodological choice to perfectly control for structural and topological variables, thereby effectively isolating the influence of seasonal changes on visual attention and psychological responses.

#### 2.1.3. Experimental Method

**Figure 2:**
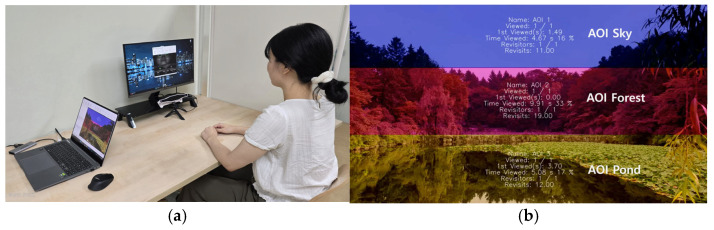
Experimental setup (**a**) and definition of areas of interest (AOIs) (**b**) (Photograph taken by the author).

The eye-tracking experiments were performed individually in a controlled indoor environment with minimal external noise and light stimulation (Figure 2). Participants were seated in front of a monitor, and three areas of the landscape (image–sky, forest, and pond) were defined as the AOI (Figure 2). All AOIs were set to the same standard for both summer and autumn images. After a 30 s visual acclimatization phase before the experiment, calibration was performed using the Gazepoint GP3 eye tracker (Gazepoint, Vancouver, BC, Canada).

During the presentation of the images, the participants observed the screen freely, and their gaze position and staring-related indicators were recorded in real time. Immediately after each stimulus was presented, the participants responded to the sense of recovery scale (PRS-11) and the SD emotional assessment. To minimize residual effects between stimuli, a rest period of 180 s was provided between images. The entire experiment lasted approximately 10–15 min per participant.

### 2.2. Measurement Scales

#### 2.2.1. Eye Tracker

A Gazepoint GP3 Eye Tracker (Gazepoint, Vancouver, BC, Canada) was used to measure gaze behavior on natural landscape images. This instrument records gaze position using non-contact corneal reflex-based estimation with a 60 Hz sampling rate and a visual angle accuracy of approximately 0.5–1.0° [18]. Adherence to standardized procedures ensured acceptable data quality [11,18]. Measurements analyses included only data with a tracking ratio of 75% or more. If the standard was not met, it was recalibrated, and if it did not improve afterwards, it was excluded from the analysis [19,20,21,22]. AOI was set to the same standard for both summer and autumn forest landscape images captured at the same location. To ensure that the images reflected the main visual elements of the natural landscape, it was divided into three AOIs, namely sky, forest, and pond, and the AOI boundaries were precisely traced along the exact physical contours separating the distinct natural elements (e.g., following the exact skyline dividing the sky and the forest and the waterline separating the forest and the pond). AOI-based gaze behavior indicators were calculated as dwell time, fixation count, and revisit count [9,18].

#### 2.2.2. PRS

PRS is a tool developed to assess how individuals perceive restorative qualities that support cognitive fatigue recovery in a given environment and is based on ART [5,23]. The shortened PRS-11 proposed by Pasini et al. [24] was used in this study. The PRS-11 consists of 11 items, including four subfactors—Being Away, Fascination, Coherence, and Scope—each of which is answered on a 5-point Likert scale, with higher scores indicating restorative perception [24]. Since PRS is a context-specific measurement that assesses restorative properties immediately after experiencing environmental stimuli [23,24], here, it was measured once for each stimulus after presenting the image of each season.

#### 2.2.3. SD Affective Evaluation

An SD-based emotional assessment was conducted to evaluate the emotional and cognitive impressions of natural landscape stimuli. SD is a technique that measures the subjective meaning of stimuli on a continuous level through bipolar adjective pairs and has been widely utilized to quantify emotional responses to environmental stimuli [25,26]. Each item is answered on a 7-point scale. Immediately after the participants were presented with images of the forest landscape in each season, they evaluated the overall impression of the stimulus. This approach can capture the emotional characteristics evoked by a landscape in a multidimensional manner and sensitively reflect differences in emotional impressions of the environment [25,27]. Recently, it has been applied to forest and natural landscape research to verify differences in emotional responses owing to seasonal changes [28].

### 2.3. Analysis

Statistical analyses were conducted using IBM SPSS Statistics (version 28.0), and Gazepoint Analysis Professional Edition eye tracking software was used for gaze data preprocessing. The analytical procedure was as follows: first, data with a tracking ratio < 75% were excluded to ensure gaze data quality. Second, a 2 × 2 mixed-design ANOVA was conducted to examine differences in psychological responses and gaze behavior by season (summer and autumn) and sex (male and female). For the ANOVA results, effect sizes were reported using partial eta squared. The total PRS and the mean SD emotional assessment scores were defined as psychological indicators, while fixation duration, fixation count, and number of revisits for each AOI were set as dependent variables. In addition, effect sizes for all ANOVA results were calculated and reported using partial eta squared.

Third, Spearman’s rho correlation analysis was performed to identify relationships between visual attention allocation and psychological recovery indicators. Furthermore, this study analyzed how gazing patterns toward seasonal landscape components interacted with emotional and cognitive responses. The statistical significance level was set at *p* = 0.05.

## 3. Results

### 3.1. Participant Characteristics and Homogeneity

A chi-square test (χ^2^ test) was conducted to verify the homogeneity of demographic characteristics between sexes used in this study. The analysis revealed no statistically significant differences between sexes in terms of age group, educational background, occupation, or residential distribution (all *p* > 0.05). These results demonstrated that the male and female groups were statistically homogeneous across major demographic variables (Table 1).

### 3.2. Psychological Reactions by Sex and Season

**Figure 3:**
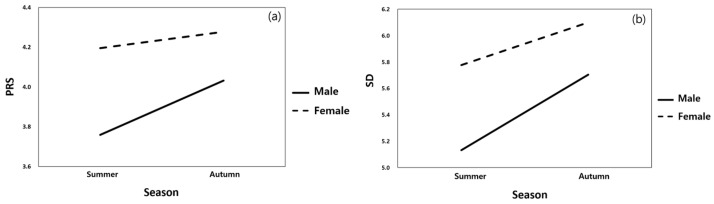
Interaction effects of sex and season on Perceived Restorativeness Scale (PRS) (**a**) and (SD) (**b**) affective evaluation.

A mixed-design ANOVA was conducted to analyze the psychological responses by sex and season (Table 2 and Table 3). The mixed-design ANOVA indicated no significant main or interaction effects for PRS, suggesting that perceived restorativeness remained relatively stable across seasons and sexes. In contrast, SD scores showed a significant main effect of sex, with females reporting consistently higher emotional evaluations than males, regardless of season. In addition, SD scores were higher in autumn than in summer for both sexes (Table 2 and Table 3; Figure 3). In particular, both males and females showed higher SD scores in autumn than in summer (Figure 3). In short, the perceived sense of recovery was stable with seasonal changes, but emotional evaluation was more positive in the fall because it was sensitive to seasonal factors.

### 3.3. AOI-Based Eye-Movement Behavior by Sex and Season

**Figure 4:**
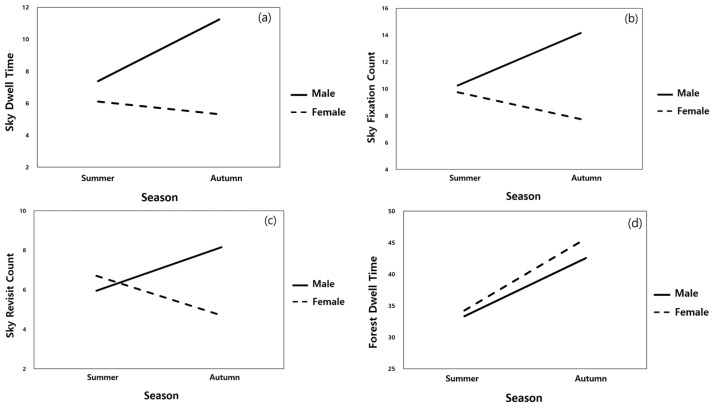

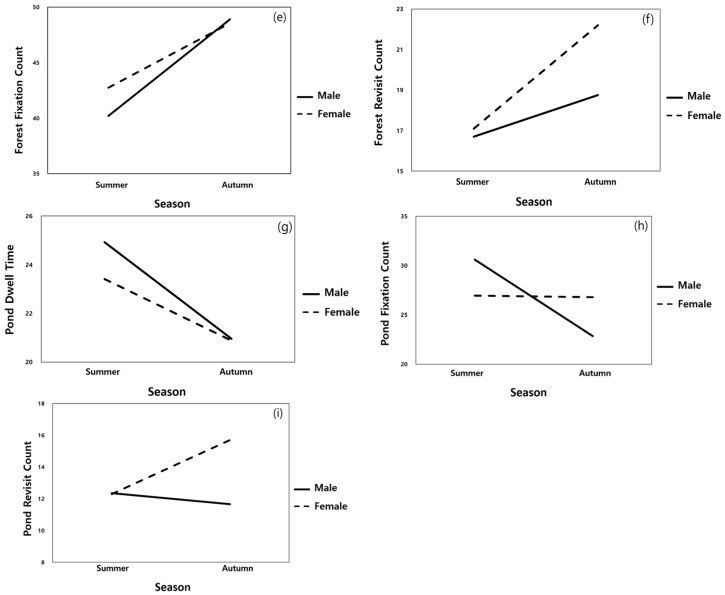
Sex and season interaction effects on eye-tracking metrics across areas of interest (sky (**a**–**c**), forest (**d**–**f**), and pond (**g**–**i**)).

Based on the AOI (sky, forest, and pond), a 2 × 2 mixed-design ANOVA was performed to analyze the differences in gaze behavior indicators by setting sex as a factor between groups and season (summer/autumn) as a repeated-measures factor (Table 4). As a result of the analysis, the interaction effect between sex and season was shown in the dwell time (F = 5.253, *p* = 0.028) and the fixation count (F = 4.380, *p* = 0.043) of the sky AOI, confirming that the distribution of attention between sexes was different depending on the season (Table 5, Figure 4). In contrast, in the forest AOI, the main effect of sex was significant for all indicators (dwell time, fixation count, and revisit count), but no interaction effect was observed (*p* > 0.05). Visual attention tended to be significantly concentrated in autumn for both sexes. The pond AOI showed no significant main effect or interaction in the main indicators but showed a tendency to approach the significance level in some indicators (*p* = 0.053–0.062) (Table 5, Figure 4). In summary, the forest AOI had a clear difference in attention distribution according to sex, whereas the sky and pond AOI induced changes in gaze behavior due to a combination of sex and seasonal factors.

### 3.4. Correlation Analysis

Spearman’s rho correlation analysis was performed to determine the relationship between the gaze behavior indicators and psychological factors (sense of recovery and emotional evaluation). In the relationship between landscape elements, visual attention to forest and pond AOI within the autumn forest landscape showed a significant negative correlation (r = −0.446, *p* < 0.01). This means that when appreciating scenery in autumn, a tradeoff occurs between the two elements. Spearman’s correlation analysis revealed a strong positive correlation (r = 0.750, *p* < 0.01) between autumn PRS and SD scores, indicating that higher aesthetic evaluations were associated with stronger restorative perceptions (Table 6).

The results confirmed that perceived recovery efficacy and emotional satisfaction in the forest environment maintained a high static association, regardless of seasonal factors. In summary, the correlation between landscape elements was heterogeneous, depending on the sky, forest, and pond, but the correlation between psychological factors showed a stable high correlation regardless of the season.

## 4. Discussion

This study comprehensively examined the restorative effects of a forest-healing landscape by integrating perceptual evaluation and AOI-based visual attention analysis. By combining eye-tracking metrics with perceived restorativeness (PRS) and semantic differential (SD) assessments, the findings contribute to a component-level understanding of how specific landscape elements influence psychological responses.

The results indicated that PRS scores remained stable across summer and autumn and between sexes, suggesting that perceived restorativeness may be grounded in the structural and contextual characteristics of the forest environment rather than in short-term seasonal visual variation [3,6]. In contrast, seasonal differences observed in SD evaluations imply that emotional impressions are more sensitive to visual attributes such as color contrast, brightness, and texture [14,16]. This pattern suggests that while cognitive restoration may depend on environmental coherence and spatial structure, affective responses are more responsive to aesthetic modulation.

Spearman’s correlation analysis revealed a strong positive correlation between autumn PRS and SD scores, indicating that higher aesthetic evaluations were associated with stronger restorative perceptions. This association between aesthetic evaluation and restorative perception can be further explained through the ‘fascination’ dimension of the Attention Restoration Theory (ART). The aesthetic attractiveness of the vibrant autumn environment engages effortless attention (soft fascination), serving as a crucial psychological mechanism that bridges positive aesthetic appraisal and cognitive restoration. However, because psychological constructs such as aesthetic evaluation and perceived restorativeness partially overlap conceptually, their relatively high correlation should be interpreted with caution and does not necessarily imply a direct causal mechanism. Nevertheless, the consistent association between PRS and SD across seasons implies that restorative perception operates as a stable psychological framework even when emotional tone varies. With respect to gaze behavior, differentiated responses to landscape elements were identified. The forest AOI attracted greater visual attention, particularly in autumn, which may reflect enhanced visual saliency during this season [28,29].

The pronounced color contrast, high saturation, and brightness of autumn foliage act as key perceptual factors that significantly impact visual preferences and aid in psychological recovery by naturally captivating top-down visual attention [30]. Therefore, the observed perceptual responses are highly dependent on this specific seasonal transformation, meaning these findings cannot be broadly generalized to forest ecosystems lacking such deciduous color shifts, such as evergreen coniferous forests [31].

The significant negative correlation between the forest and pond AOIs in autumn indicates a competitive allocation of perceptual resources. Specifically, the vivid and contrasting leaf colors of the autumn forest strongly drew the observers’ visual attention toward the canopy, competitively reducing fixation on the water body. Interestingly, the marginally significant indicators observed in the pond AOI imply that water elements consistently retain visual attractiveness, even when competing with highly salient seasonal stimuli. Seasonal variation, therefore, appears to reorganize attentional strategies rather than simply amplify overall attention.

Regarding sex-related differences, the significant interaction effects between season and sex observed in the sky AOI suggest that males and females may employ distinct perceptual or exploratory strategies when viewing open spaces [32,33,34]. However, the theoretical explanation for these differences in visual exploration remains largely exploratory. Given the relatively small sample size of this study, it is crucial to consider alternative explanations, such as sampling variability. Consequently, these findings regarding sex differences should be interpreted with caution.

From a theoretical perspective, the present findings suggest that restorative perception may remain structurally stable across seasonal changes, whereas affective evaluations are more sensitive to visual variation. This distinction implies that cognitive restoration and emotional appraisal, although interrelated, may operate through partially distinct perceptual pathways. Practically, the results provide implications for forest-healing landscape design. Given that forest components elicited greater visual attention and more positive emotional responses in autumn, seasonal planting strategies emphasizing color contrast and textural diversity may enhance user engagement. Despite these contributions, several limitations of this study must be noted. First, the most critical limitation is the extremely small number of experimental stimuli (N = 2), which, combined with a relatively small sample size, substantially restricts the generalizability of the results to broader forest environments. Because only two specific photographs were used, it is difficult to definitively disentangle true seasonal effects from the specific visual characteristics of those images (e.g., color composition and spatial structure) [31,35].

In particular, the two images differed substantially in the appearance of the sky; the autumn image featured a pronounced contrast between the blue sky and clouds, whereas the summer image exhibited uniform white-gray cloudiness. Because visual salience and luminance contrast can involuntarily capture visual attention, this difference in sky appearance may have acted as a confounding variable influencing gaze allocation [36].

Therefore, the observed differences in eye movements cannot be solely attributed to seasonal foliage changes. Second, the indoor eye-tracking experiment using static images could not fully replicate the dynamic, multi-sensory experience of a real forest. Third, participants’ subjective expectations and their baseline frequencies of nature contact were not rigorously controlled, which might have influenced their psychological evaluations. Considering these constraints, the correlations between psychological and eye-tracking measures should be interpreted with caution, and the present findings are explicitly framed as a preliminary exploratory study. Future research should utilize a larger set of stimuli and incorporate immersive technologies, such as virtual reality (VR), along with nature contact frequency as a control variable, to provide a more comprehensive understanding of restorative mechanisms [30,31,37].

## 5. Conclusions

This study investigated seasonal and sex-related differences in psychological restoration and visual attention within a forest-healing landscape by integrating perceived restorativeness (PRS), emotional evaluation (SD), and AOI-based eye-tracking measures. The findings demonstrated that perceived restorativeness remained relatively stable across seasons and sexes, whereas emotional evaluations were more sensitive to seasonal visual variation. In particular, autumn landscapes elicited stronger aesthetic impressions and greater visual attention toward forest components.

The strong association between PRS and SD scores suggests that aesthetic appreciation and restorative perception are closely interconnected, although they may respond differently to structural versus surface-level environmental features. Moreover, seasonal shifts in gaze allocation indicate that attentional strategies are dynamically reorganized according to visual saliency rather than uniformly intensified.

From a theoretical standpoint, the results suggest a distinction between structurally stable restorative perception and seasonally responsive affective appraisal, implying partially differentiated perceptual pathways within the restorative process. Practically, the findings highlight the importance of considering seasonal aesthetics and user characteristics in forest-healing landscape design and management. To enhance the practical applicability of these findings, we propose operational landscape design schemes for forest-healing environments. First, to minimize visual competition during peak foliage seasons, landscape architects should reasonably lay out forest and water elements by planting single-green evergreen shrubs as visual buffers between the vibrant autumn foliage and the water’s edge or by designing separated trails that sequentially reveal water features and colorful canopies [37,38]. Second, to optimize the configuration of landscape visual elements for different genders—given their distinct visual exploration strategies toward open spaces (sky and pond)—designers should offer diverse viewing options. This can be achieved by alternating expansive, open viewpoints (Prospect) with enclosed, vegetation-screened seating areas (Refuge) along the waterfront, thereby accommodating the varied perceptual preferences of both males and females.

Although the study was limited to static images from a single forest-healing site and should be interpreted as a preliminary exploratory study, the integrated methodological approach provides a foundation for future research incorporating immersive environments and physiological indicators. Continued investigation across diverse forest types and user groups will further clarify the mechanisms underlying seasonal variation in restorative experience.

## Figures and Tables

**Table 1:** Demographic characteristics of participants and homogeneity tests by sex.

Variable	Category	Sex		Total (%)	χ^2^	*p*-Value	
		Male (%)	Female (%)				
Age group	40 s	5 (25.0)	8 (40.0)	13 (32.5)	2.563	0.278	
	50 s	4 (20.0)	6 (30.0)	10 (25.0)			
	60 s	11 (55.0)	6 (30.0)	17 (42.5)			
Education	University student	10 (50.0)	9 (45.0)	19 (47.5)	0.144	0.931	
	graduate school student	5 (25.0)	6 (30.0)	11 (27.5)			
	graduate school graduate	5 (25.0)	5 (25.0)	10 (25.0)			
Occupation	Office worker	11 (55.0)	5 (25.0)	16 (40.0)	9.393	0.094	
	Self-employed	1 (5.0)	5 (25.0)	6 (15.0)			
	Student	2 (10.0)	1 (5.0)	3 (7.5)			
	Professional	4 (20.0)	3 (15.0)	7 (17.5)			
	Public servant	2 (10.0)	2 (10.0)	4 (10.0)			
	Housewife	0 (0.0)	4 (20.0)	4 (10.0)			
Residence	Gangwon Province	20 (100.0)	20 (100.0)	40 (100.0)	-		-

**Table 2:** Results of the mixed-design ANOVA for psychological responses by sex and season.

Measure	Source	Sum of Squares	df	Mean Square	F	*p*-Value	Partial Eta-Squared
PRS	Sex	0.629	1.000	0.629	1.762	0.192	0.044
	Sex × Season	0.182	1.000	0.182	0.511	0.479	0.013
	Error	13.553	38.000	0.357			
SD	Sex	4.009	1.000	4.009	11.503	0.002	0.232
	Sex × Season	0.313	1.000	0.313	0.897	0.350	0.023
	Error	13.244	38.000	0.349			

**Table 3:** Descriptive statistics of perceived restorativeness and semantic differential (SD) by sex and season.

Measure	Season	Sex	Mean	Standard Error
PRS	Summer	Male	3.759 ^a^	0.142
		Female	4.195 ^b^	0.142
	Autumn	Male	4.032 ^a^	0.181
		Female	4.277 ^b^	0.181
SD	Summer	Male	5.132 ^a^	0.195
		Female	5.777 ^b^	0.195
	Autumn	Male	5.705 ^a^	0.225
		Female	6.100 ^b^	0.225

Bonferroni: a < b (Superscript letters (a, b) denote the results of Bonferroni post hoc comparisons. Means sharing different letters are significantly different (*p* < 0.05), with higher values indicated by subsequent letters).

**Table 4:** Results of the mixed-design ANOVA for area-of-interest (AOI)-based eye-tracking metrics by sex and season.

Measure	Source	Sum of Squares	df	Mean Square	F	*p*-Value	Partial Eta-Squared
Sky AOI Dwell Time	Sex	46.575	1.000	46.575	2.261	0.141	0.056
	Sex × Season	108.180	1.000	108.180	5.253	0.028	0.121
	Error	782.626	38.000	20.595			
Sky AOI Fixation Count	Sex	18.050	1.000	18.050	0.454	0.504	0.012
	Sex × Season	174.050	1.000	174.050	4.380	0.043	0.103
	Error	1509.900	38.000	39.734			
Sky AOI Revisit Count	Sex	0.200	1.000	0.200	0.008	0.927	0.000
	Sex × Season	88.200	1.000	88.200	3.738	0.061	0.090
	Error	896.600	38.000	23.595			
Forest AOI Dwell Time	Sex	2135.552	1.000	2135.552	12.250	0.001	0.244
	Sex × Season	24.534	1.000	24.534	0.141	0.710	0.004
	Error	6624.542	38.000	174.330			
Forest AOI Fixation Count	Sex	1051.250	1.000	1051.250	7.977	0.008	0.174
	Sex × Season	42.050	1.000	42.050	0.319	0.575	0.008
	Error	5007.700	38.000	131.782			
Forest AOI Revisit Count	Sex	255.612	1.000	255.612	7.430	0.010	0.164
	Sex × Season	46.513	1.000	46.513	1.352	0.252	0.034
	Error	1307.375	38.000	34.405			
Pond AOI Dwell Time	Sex	210.474	1.000	210.474	1.320	0.258	0.034
	Sex × Season	10.248	1.000	10.248	0.064	0.801	0.002
	Error	6058.477	38.000	159.434			
Pond AOI Fixation Count	Sex	312.050	1.000	312.050	3.996	0.053	0.095
	Sex × Season	288.800	1.000	288.800	3.699	0.062	0.089
	Error	2967.150	38.000	78.083			
Pond AOI Revisit Count	Sex	36.450	1.000	36.450	1.703	0.200	0.043
	Sex × Season	84.050	1.000	84.050	3.926	0.055	0.094
	Error	813.500	38.000	21.408			

**Table 5:** Descriptive statistics of area-of-interest (AOI)-based eye-tracking metrics by sex and season.

Measure	Season	Sex	Mean	Standard Error
Sky AOI Dwell Time	Summer	Male	7.383 ^b^	1.640
		Female	6.111 ^a^	1.640
	Autumn	Male	11.235 ^b^	1.121
		Female	5.311 ^a^	1.121
Sky AOI Fixation Count	Summer	Male	10.250 ^b^	2.263
		Female	9.750 ^a^	2.263
	Autumn	Male	14.150 ^b^	1.747
		Female	7.750 ^a^	1.747
Sky AOI Revisit Count	Summer	Male	5.950 ^a^	1.500
		Female	6.700 ^b^	1.500
	Autumn	Male	8.150 ^b^	1.234
		Female	4.700 ^a^	1.234
Forest AOI Dwell Time	Summer	Male	33.325 ^a^	3.537
		Female	34.225 ^b^	3.537
	Autumn	Male	42.551 ^a^	3.161
		Female	45.666 ^b^	3.161
Forest AOI Fixation Count	Summer	Male	40.200 ^a^	3.291
		Female	42.750 ^b^	3.291
	Autumn	Male	48.900 ^b^	2.318
		Female	48.550 ^a^	2.318
Forest AOI Revisit Count	Summer	Male	16.700 ^a^	1.458
		Female	17.100 ^b^	1.458
	Autumn	Male	18.750 ^a^	1.253
		Female	22.200 ^b^	1.253
Pond AOI Dwell Time	Summer	Male	24.922 ^b^	3.460
		Female	23.412 ^a^	3.460
	Autumn	Male	20.962 ^b^	3.003
		Female	20.883 ^a^	3.003
Pond AOI Fixation Count	Summer	Male	30.600 ^b^	3.260
		Female	22.850 ^a^	3.046
	Autumn	Male	26.950 ^b^	3.260
		Female	26.800 ^a^	3.046
Pond AOI Revisit Count	Summer	Male	12.350 ^b^	1.594
		Female	12.300 ^a^	1.594
	Autumn	Male	11.650 ^a^	1.093
		Female	15.700 ^b^	1.093

Bonferroni: a < b (Superscript letters (a, b) denote the results of Bonferroni post hoc comparisons. Means sharing different letters are significantly different (*p* < 0.05), with higher values indicated by subsequent letters).

**Table 6:** Spearman’s rho correlation coefficients between eye-tracking metrics and psychological responses.

Variable	1	2	3	4	5	6	7	8	9	10
1. Summer Sky AOI	1									
2. Summer Forest AOI	0.122	1								
3. Summer Pond AOI	−0.415 **	−0.309	1							
4. Autumn Sky AOI	0.547 **	−0.223	−0.234	1						
5. Autumn Forest AOI	−0.188	0.099	−0.047	−0.207	1					
6. Autumn Pond AOI	−0.162	0.111	0.439 **	−0.107	−0.446 **	1				
7. Summer PRS	0.180	0.009	0.115	−0.130	−0.110	0.032	1			
8. Autumn PRS	0.184	−0.085	0.175	0.009	0.196	0.125	0.581 **	1		
9. Summer SD	0.157	−0.023	0.233	−0.126	−0.206	0.145	0.507 **	0.306	1	
10. Autumn SD	0.101	0.043	0.143	−0.004	0.098	0.304	0.398 *	0.750 **	0.533 **	1

Area of interest: AOI; * *p* < 0.05; ** *p* < 0.01.

## Data Availability

The data presented in this study are not publicly available due to privacy and ethical restrictions in accordance with the approved Institutional Review Board (IRB) protocol. Data are available from the corresponding author upon reasonable request and subject to IRB approval.

## References

[1] Miller J.R. (2005). Biodiversity conservation and the extinction of experience. Trends Ecol. Evol..

[2] Twohig-Bennett C., Jones A. (2018). The health benefits of the great outdoors: A systematic review and meta-analysis of greenspace exposure and health outcomes. Environ. Res..

[3] Hartig T., Evans G.W., Jamner L.D., Davis D.S., Gärling T. (2003). Tracking restoration in natural and urban field settings. J. Environ. Psychol..

[4] Bratman G.N., Hamilton J.P., Hahn K.S., Daily G.C., Gross J.J. (2015). Nature experience reduces rumination and subgenual prefrontal cortex activation. Proc. Natl. Acad. Sci. USA.

[5] Kaplan S. (1995). The restorative benefits of nature: Toward an integrative framework. J. Environ. Psychol..

[6] Kaplan R., Kaplan S. (1989). The Experience of Nature: A Psychological Perspective.

[7] Ulrich R.S. (1984). View through a window may influence recovery from surgery. Science.

[8] Berto R. (2005). Exposure to restorative environments helps restore attentional capacity. J. Environ. Psychol..

[9] Duchowski A.T., Duchowski A.T. (2017). Eye Tracking Methodology: Theory and Practice.

[10] Dupont L., Van Eetvelde V. (2014). Eye-tracking analysis in landscape perception research: Influence of photograph properties and landscape characteristics. Landsc. Res..

[11] Nordh H., Hagerhall C.M., Holmqvist K. (2013). Tracking restorative components: Patterns in eye movements as a consequence of a restorative rating task. Landsc. Res..

[12] Berto R., Baroni M.R., Zainaghi A., Bettella S. (2010). An exploratory study of the effect of high and low fascination environments on attentional fatigue. J. Environ. Psychol..

[13] Fleming W., Rizowy B., Shwartz A. (2024). The nature gaze: Eye-tracking experiment reveals well-being benefits derived from directing visual attention towards elements of nature. People Nat..

[14] Lee J., Park B.J., Tsunetsugu Y., Kagawa T., Miyazaki Y. (2009). Restorative effects of viewing real forest landscapes, based on a comparison with urban landscapes. Scand. J. For. Res..

[15] Lee Y.H., Park C.W., Ha S.Y. (2015). A study on the image and visual preference for the beautiful forest scenery types in Korea. J. Korean For. Soc..

[16] Ulrich R.S., Altman I., Wohlwill J.F. (1983). Aesthetic and affective response to natural environment. Human Behavior and Environment.

[17] White M.P., Smith A., Humphryes K., Pahl S., Snelling D., Depledge M.H. (2010). Blue space: The importance of water for preference, affect, and well-being. J. Environ. Psychol..

[18] Holmqvist K., Nyström M., Andersson R., Dewhurst R., Jarodzka H., van de Weijer J. (2011). Eye Tracking: A Comprehensive Guide to Methods and Measures.

[19] Chamberlain L. (2007). Eye Tracking Methodology: Theory and Practice. Qual. Mark. Res. Int. J..

[20] Dalmaijer E.S., Mathôt S., Van der Stigchel S. (2014). PyGaze: An open-source, cross-platform toolbox for minimal-effort programming of eye tracking experiments. Behav. Res. Methods.

[21] Orquin J.L., Holmqvist K. (2018). Threats to the validity of eye-movement research in psychology. Behav. Res. Methods.

[22] Poole A., Ball L.J., Ghaoui C. (2006). Eye tracking in HCI and usability research. Encyclopedia of Human Computer Interaction.

[23] Hartig T., Korpela K., Evans G.W., Gärling T. (1997). A measure of restorative quality in environments. Scand. Hous. Plan. Res..

[24] Pasini M., Berto R., Scopelliti M., Carrus G. (2009). Measuring the Restorative Value of the Environment: Contribution to the Validation of the Italian Version of the Perceived Restorativeness Scale.

[25] Osgood C.E., Suci G.J., Tannenbaum P.H. (1957). The Measurement of Meaning.

[26] Russell J.A., Pratt G. (1980). A description of the affective quality attributed to environments. J. Pers. Soc. Psychol..

[27] Küller R., Gärling T., Evans G.W. (1991). Neuropsychological perspective. Environment, Cognition, and Action: An Integrated Approach.

[28] Nordh H., Hartig T., Hagerhall C.M., Fry G. (2009). Components of small urban parks that predict the possibility for restoration. Urban For. Urban Green..

[29] Berman M.G., Jonides J., Kaplan S. (2008). The cognitive benefits of interacting with nature. Psychol. Sci..

[30] Yin M., Li K., Xu Z., Jiao R., Yang W. (2024). Exploring the impact of autumn color and bare tree landscapes in virtual environments on human well-being and therapeutic effects across different sensory modalities. PLoS ONE.

[31] Lin W., Mu Y., Zhang Z., Wang J., Diao X., Lu Z., Guo W., Wang Y., Xu B. (2022). Research on cognitive evaluation of forest color based on visual behavior experiments and landscape preference. PLoS ONE.

[32] Jiang G., Cao S., Chen S., Tian X., Cao M. (2025). Gender Differences in Visual Perception of Park Landscapes Based on Eye-Tracking Technology: A Case Study of Beihai Park in Beijing. Buildings.

[33] Grady C.L., McIntosh A.R., Horwitz B. (1995). Age-related reductions in human recognition memory due to impaired encoding. Science.

[34] Völker S., Kistemann T. (2011). The impact of blue space on human health and well-being—Salutogenetic health effects of inland surface waters: A review. Int. J. Hyg. Environ. Health.

[35] Kardan O., Demiralp E., Hout M.C., Hunter M.R., Karimi H., Hanayik T., Yourganov G., Jonides J., Berman M.G. (2015). Is the preference of natural versus man-made scenes driven by bottom–up processing of the visual features of nature?. Front. Psychol..

[36] Neilson B.N., Craig C.M., Travis A.T., Klein M.I. (2019). A review of the limitations of Attention Restoration Theory and the importance of its future research for the improvement of well-being in urban living. Vis. Sustain..

[37] Xing G., Gao F., Tang J., Dong B., Zhang X. (2025). The Differences of Emotional Restoration Effects of Colored-Leaves Plant Communities in Urban Parks. Landsc. Archit. Front..

[38] Wu L., Zhang Y., Mao M., Li C., Zhang Q., Zhao W., Sui X., Li J., Ma J., Li Y. (2024). Evergreen or seasonal? Quantitative research on the color of urban scenic forests based on stress—Attention electroencephalogram feedback. Front. For. Glob. Change.

